# Supplementary material for: p140Cap inhibits β-Catenin in the breast cancer stem cell compartment instructing a protective anti-tumor immune response
Source: Nat Commun. 2023 May 11;14:2350. doi: 10.1038/s41467-023-37824-y (PMC10175288; doi:10.1038/s41467-023-37824-y)
Supplement: Supplementary file 1 — Supplementary Information [file 41467_2023_37824_MOESM1_ESM.pdf]

Supplementary Information

p140Cap inhibits β-Catenin in the breast cancer stem cell compartment instructing a protective anti-tumor immune response

Vincenzo Salemme[1,2], Mauro Vedelago[1], Alessandro Sarcinella[1], Federico Moietta[1], Alessio Piccolantonio[1,2], Enrico Moiso[1], Giorgia Centonze[1,2], Marta Manco[1], Andrea Guala[1], Alessia Lamolinara[3], Costanza Angelini[1], Alessandro Morellato[1,2], Dora Natalini[1], Raffaele Calogero[1,2], Danny Incarnato[4], Salvatore Oliviero[2,5], Laura Conti[1,2], Manuela Iezzi[3], Daniela Tosoni[6], Giovanni Bertalot[6], Stefano Freddi[6], Francesco A. Tucci[6,7], Francesco De Santis[8], Cristina Frusteri[8], Stefano Ugel[8], Vincenzo Bronte[8], Federica Cavallo[1,2], Paolo Provero[9], Marta Gai[1], Daniela Taverna[1,2], Emilia Turco[1], Salvatore Pece[6,10*] and Paola Defilippi[1,2**].

[1]Department of Molecular Biotechnology and Health Sciences, University of Torino, Via Nizza 52, 10126, Torino, Italy.
[2]Molecular Biotechnology Center (MBC) "Guido Tarone", Via Nizza, 52, 10126, Turin, Italy
[3]Immuno-Oncology Laboratory, Center for Advanced Studies and Technologies; Department of Neurosciences, Imaging and Clinical Sciences. G. d'Annunzio University of Chieti-Pescara, Italy
[4]Department of Molecular Genetics, Groningen Biomolecular Sciences and Biotechnology Institute (GBB), University of Groningen, Groningen, the Netherlands.
[5]Department of Life Sciences and Systems Biology, University of Turin, Torino, Italy.
[6]European Institute of Oncology IRCCS, 20141 Milan, Italy
[7]School of Pathology, University of Milan, Milan, Italy.
[8]Immunology Section, Department of Medicine, University of Verona, 37134 Verona, Italy
[9]Neuroscience Department "Rita Levi Montalcini", University of Torino, Via Cherasco 15, 10126 Torino, Italy.
[10]Department of Oncology and Hemato-Oncology, Università degli Studi di Milano, 20142 Milano, Italy.

**Corresponding Author: Paola Defilippi (paola.defilippi@unito.it)
*Co-corresponding Author: Salvatore Pece (salvatore.pece@ieo.it)

ALL patients

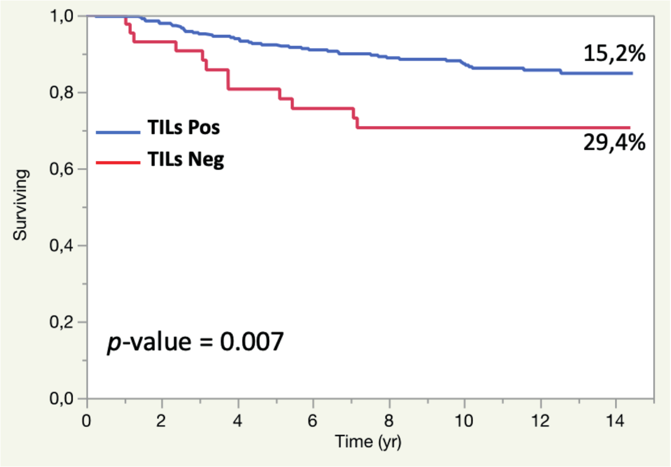

	TILs Neg	TILs Pos	Total
p140Cap Low	15 (36,5%)	66 (20,4%)	81
p140Cap High	26 (63.4%)	257 (79,5%)	283
Total	41	323	364

OR = 2,25 (1,13;4,48)
P = 0,02

HER2⁻ patients

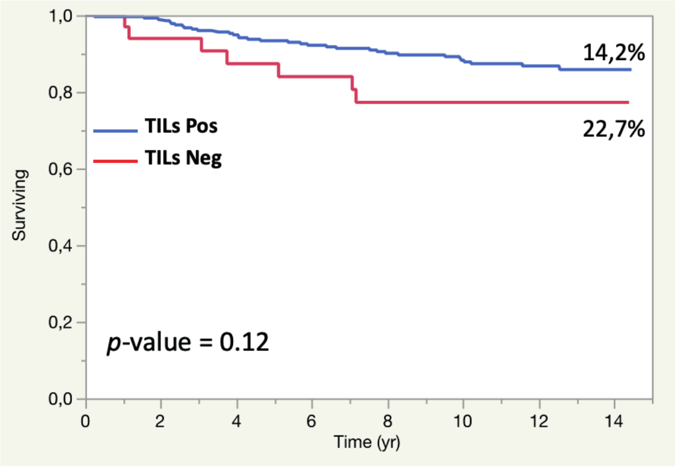

	TILs Neg	TILs Pos	Total
p140Cap Low	11 (29,1%)	51 (19,0%)	62
p140Cap High	21 (65,6%)	217 (80,9%)	238
Total	32	268	300

OR = 2,23 (1,01;4,91)
P = 0,04

HER2⁺ patients

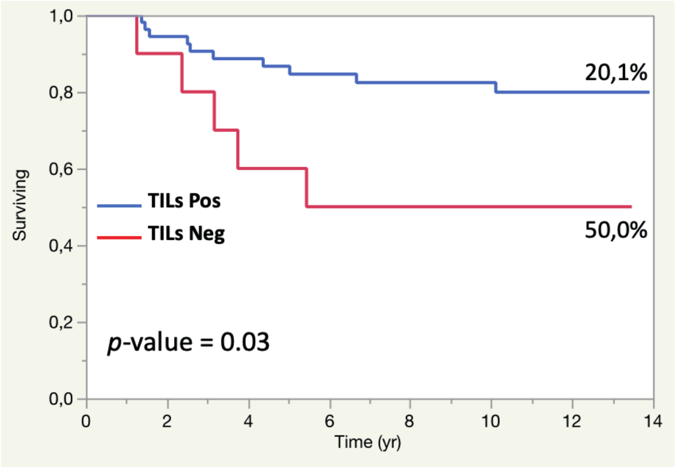

	TILs Neg	TILs Pos	Total
p140Cap Low	4 (44,4%)	15 (27,2%)	19
p140Cap High	5 (55,5%)	40 (72,7%)	45
Total	9	55	64

OR = 2,13 (0,50;9,03)
P = 0,30

Supplementary Fig. 1

Left, The overall survival rates of the negative and positive stromal TIL groups were analyzed using Kaplan-Meier survival analysis, and the statistical significance of between-group differences was evaluated using the Log-rank Test. Univariate and multivariate Cox regression analyses were performed to identify the prognostic significance of stromal TILs. Right, Analysis of the distribution of TIL-positive and TIL-negative female patients according to their p140Cap status (p140Cap^{HIGH} vs. p140Cap^{LOW}) in all patients (top) and in the subgroup of HER2-negative (middle) and HER2-positive (bottom) patients; p-value, Pearson's Chi-Squared Test.

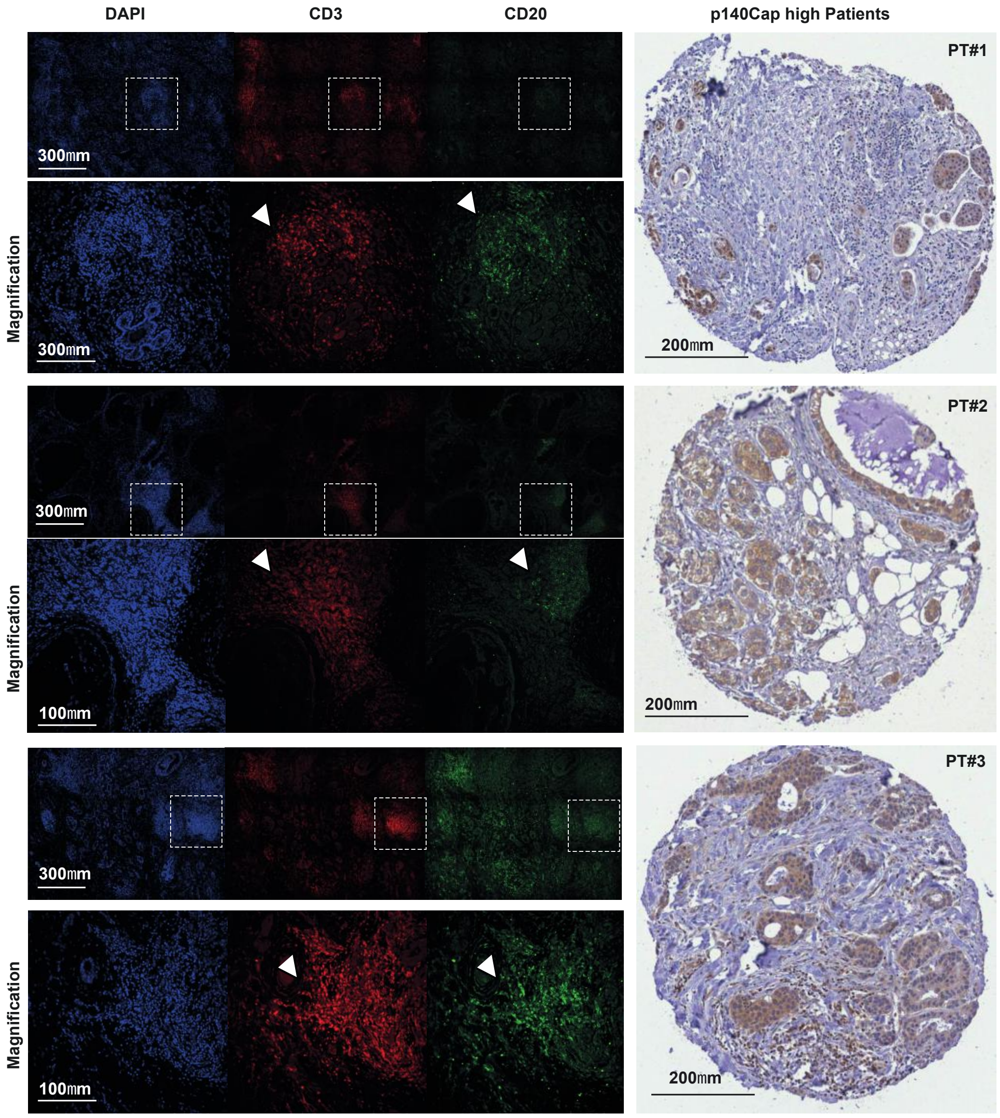

Supplementary Fig. 2

Left, Representative immunofluorescence images from the immunophenotypic characterization of the TIL infiltrate in n=3 different p140Cap^{HIGH} breast tumors performed on 3μm-thick FFPE sections using an anti-CD3 (red) and anti-CD20 (green) antibody to recognize T- and B-lymphocytes, respectively. For each tumor sample (n=3), a tile merge (top panels) of 12 representative regions of interest (ROI) acquired independently is shown for each staining (blu, DAPI; red, CD3; green, CD20). A representative ROI from the tile merge for each staining is magnified in bottom panels. Right, Representative immunohistochemistry images of the TMA cores of the three different p140Cap^{HIGH} breast tumors (PT#1-3). Scale bars are indicated.

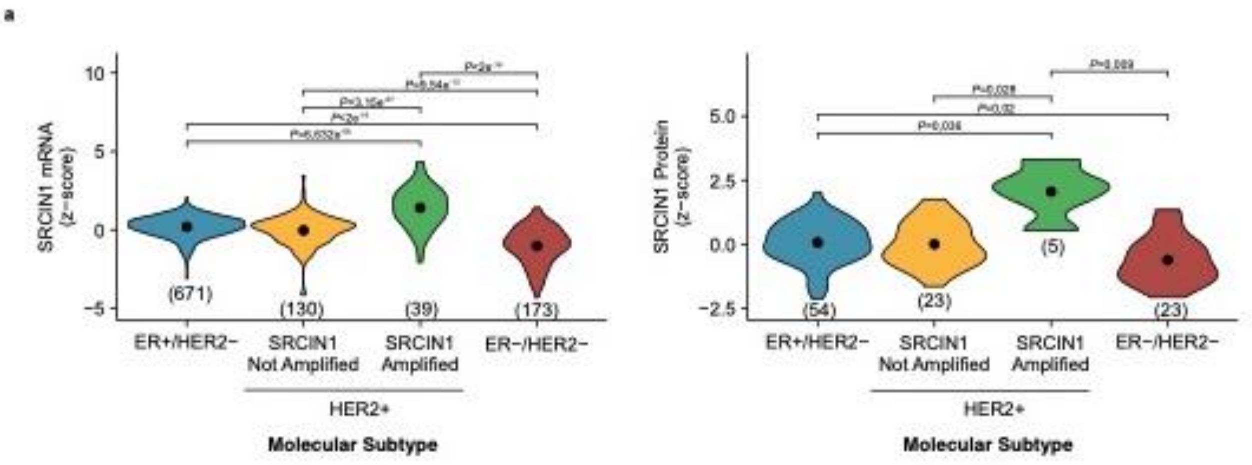

b

Correlation with p140Cap and molecular subtypes

	p140Cap High	p140Cap Low	Total	OR (CI)	p
ER+/HER2-	325 (80.7%)	78 (19.3%)	403	Reference	
HER2+	45 (67.2%)	22 (32.8%)	67	2.04 (1.16-3.59)	0.012
ER-/HER2-	16 (57.1%)	12 (42.9%)	28	3.13 (1.42-6.87)	0.0031
Total	386	112	498		

Supplementary Fig. 3

a Analysis of p140Cap expression across the different molecular subtypes of human BC. Violin plots show the expression levels of *SRCIN1* transcripts (left) and p140Cap protein (right) in different molecular subtypes of human BC patients from the 1095 BC female patients from TCGA-BRCA cohort [34]. The black dots indicate the average. The number of patients for each subtype is shown in parentheses. For the HER2+ subtype, tumors with presence or absence of *SRCIN1* co-amplification are shown.

b Analysis of the distribution of p140Cap^{HIGH} and p140Cap^{LOW} female patients of the 498 BC female patients of the IEO cohort, see Methods, according to the different molecular subtypes: luminal (ER+/HER2-), HER2-amplified (HER2+) and triple-negative (ER-/HER2-); Odds Ratio (OR) with 95% Confidence Intervals (CI) are indicated; p-value, Pearson's Chi-Squared Test.

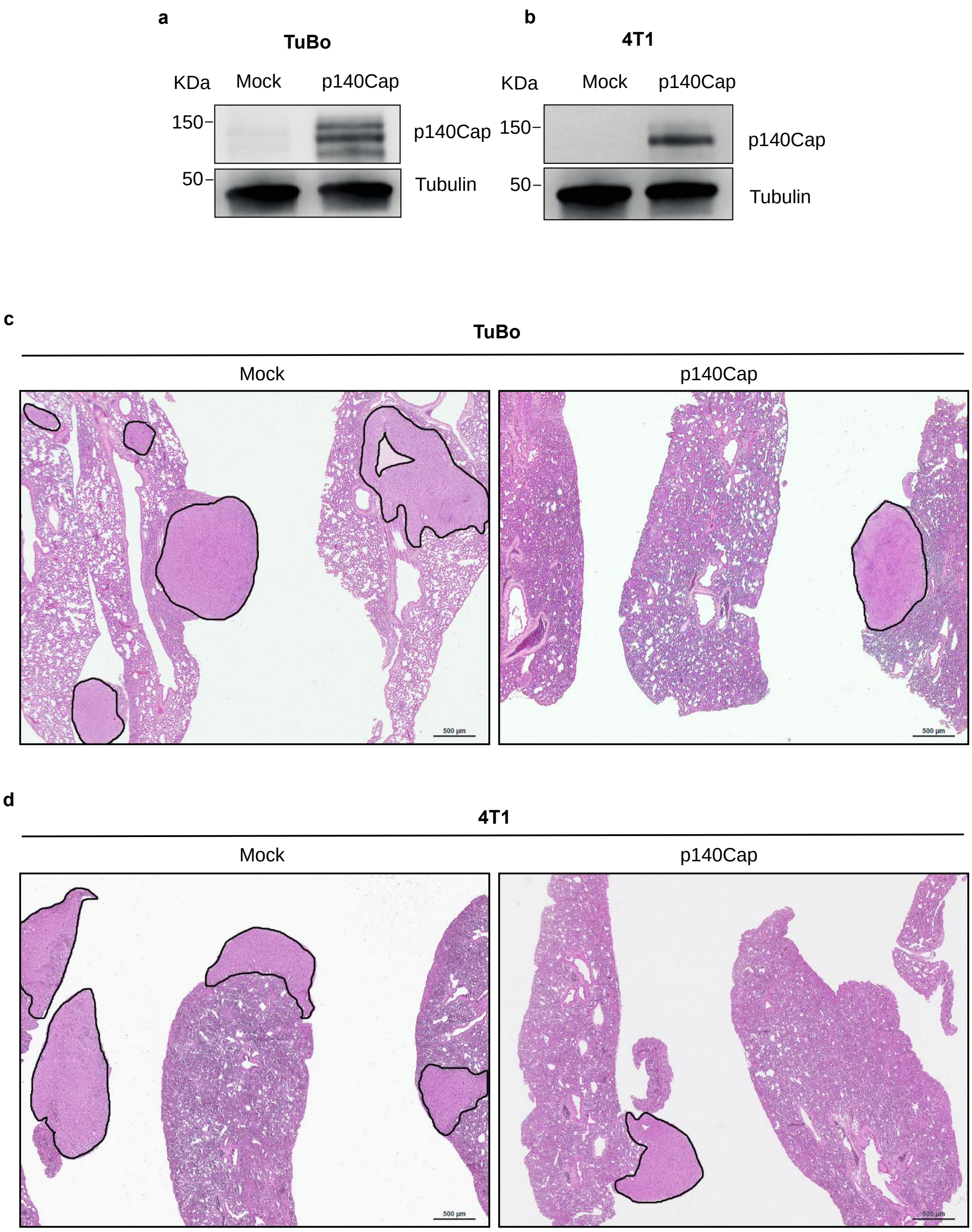

Supplementary Fig. 4

a-b Western Blot analysis of a pool of four clones with enforced p140Cap expression upon retroviral infection in TuBo in **a**, and 4T1 cells in **b** (n=10 experimental repeats). Tubulin was used as loading control.

c-d A set of representative H&E images of lung tissues from mice injected with mock or p140Cap TuBo, or 4T1 cells, where each figure shows the metastatic area (see also Methods); TuBo, n=5 mice; 4T1, n= 10 mice.

.

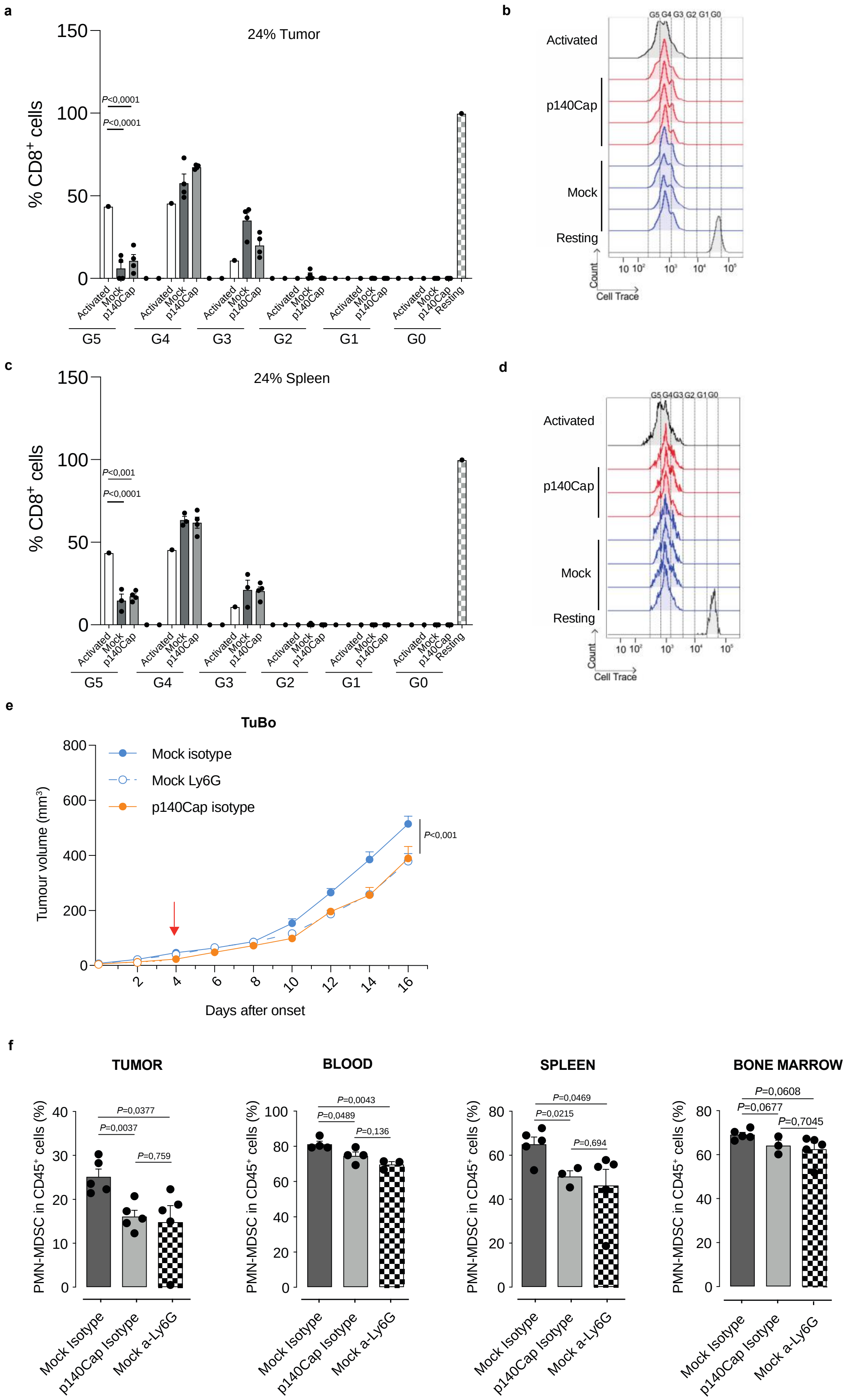
a
24% Tumor
% CD8+ cells
150
100
50
0
P<0,0001
P<0,0001
Activated
Mock
p140Cap
Resting
G5
G4
G3
G2
G1
G0
b
Activated
p140Cap
Mock
Resting
G5 G4 G3 G2 G1 G0
Count
Cell Trace
c
24% Spleen
% CD8+ cells
P<0,001
P<0,0001
d
e
TuBo
Mock isotype
Mock Ly6G
p140Cap isotype
Tumour volume (mm3)
800
600
400
200
0
P<0,001
Days after onset
2
4
6
8
10
12
14
16
f
TUMOR
BLOOD
SPLEEN
BONE MARROW
PMN-MDSC in CD45+ cells (%)
P=0,0377
P=0,0037
P=0,759
P=0,0043
P=0,0489
P=0,136
P=0,0469
P=0,0215
P=0,694
P=0,0608
P=0,0677
P=0,7045
Mock Isotype
p140Cap Isotype
Mock a-Ly6G

87

Supplementary Fig. 5

Suppressive activity of tumor-infiltrating CD11b+ cells isolated from either p140Cap (red) or Mock (blue) tumor-bearing mice co-cultured with activated T cells in presence, was evaluated by flow cytometry tracking cell trace dilution in Cell Trace-labelled; peptide activated HA-specific T cells after 3 days.

a Cell Trace dilution representative plots of CTLs co-cultured with 24% of tumor-isolated CD11b+ cells are shown. Data are represented for n=4 mice/group as mean ± SEM; two-tailed unpaired *t* test.

b T cell proliferative generations (from G0 to G5) under resting condition (not activated by peptide, grey line) or stimulated and co-cultured with CD11b+ cells derived from Mock-derived (blues) or p14Cap (red) tumors. Data are represented for n=4 mice/group as mean ± SEM; 2way ANOVA test. G: generations. Suppressive activity of splenic Ly6G+ cells isolated from either p140Cap (red) or Mock (blue) tumor-bearing mice co-cultured with activated T cells in presence was evaluated by flow cytometry tracking cell trace dilution in Cell Trace-labelled; peptide activated HA-specific T cells after 3 days.

c Cell trace dilution representative plots of CTLs co-cultured with 24% of splenic Ly6G+ cells. (D) T cell proliferative generations (from G0 to G5) under resting condition (not activated by peptide, grey line) or stimulated and co-cultured with Ly6G+ cells derived from Mock-derived (blues) or p140Cap (red) tumors. Data are represented for n=4 mice/group as mean ± SEM; 2way ANOVA test.

e-f In vivo anti-Ly6G treatment.

e 10^5 TuBo Mock and p140Cap cells were injected into the mammary fat pad of female BALB/c mice. When tumors size reached 80 mm3, anti-Ly6G antibody treatment was performed by two IP injections per week, of 100 μg of anti-Ly6G (n=5) or control IgG2a isotype antibodies (n=5/Mock isotype; n=4/p140Cap isotype). Tumor growth was monitored and tumor size was measured. Data are represented for n=5 mice/group as mean ± SEM; 2way ANOVA test.

f Flow cytometry analysis for PMN-MDSCs. Representative flow cytometry bar plots showed the percentage (%) of PMN-MDSC (CD45+CD11b+Ly6G+Ly6Clow) cells normalised on CD45+ cells, in tumor (n=5), blood (n=4/Mock isotype; n=4/p140Cap isotype; n=3/Mock anti-Ly6G), spleen (n=5/Mock isotype; n=3/p140Cap isotype; n=5/Mock anti-Ly6G) and bone marrow (n=5/Mock isotype; n=3/p140Cap isotype; n=5/Mock anti-Ly6G) of mice treated as in (E) with anti-Ly6G and anti-isotype administrations. Data are represented for n=x mice/group as mean ± SEM; two-tailed unpaired t test.

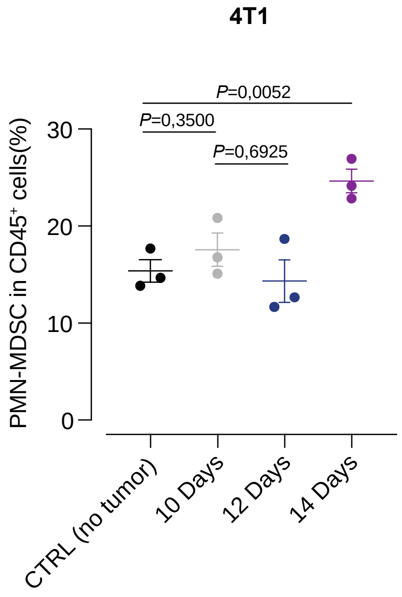

Supplementary Fig. 6

FACS analysis of PMN-MDSCs (CD45+CD11b+Ly6G+Ly6Clow) cells normalised on CD45+ cells in lungs of Mock 4T1 tumor-bearing mice after 10, 12 and 14 days post-injection. Data are represented for n=3 mice/group; dot plot represented as mean ± SEM; two-tailed unpaired t test.

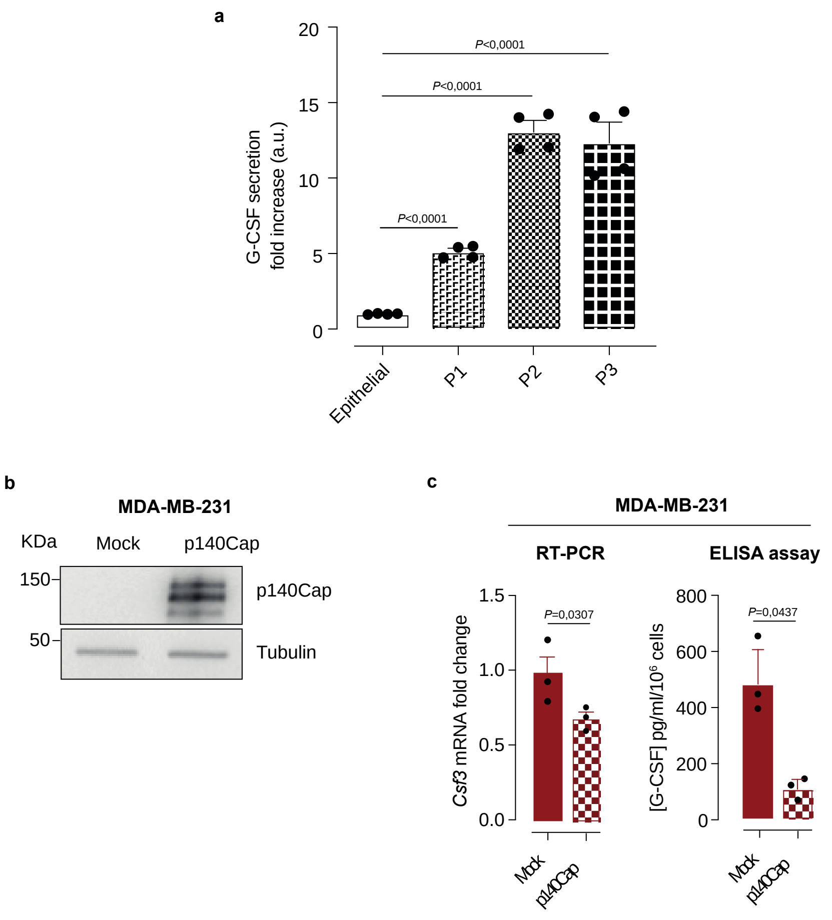

Supplementary Fig. 7

a ELISA assay of G-CSF secretion in Mock TUBO cell culture supernatants and in P1, P2 and P3 mammosphere supernatants. Data are represented for n=4 experimental repeats; bar plot represented G-CSF fold increase as mean ± SEM; two-tailed unpaired t test.

b Generation of MDA-MB-231 p140Cap overexpressing cells. Western Blot analysis of a pool of four clones of MDA-MB-231 cells expressing p140Cap upon retroviral infection. Data are represented for n=10 experimental repeats. Tubulin was used as loading control.

c G-CSF transcript and G-CSF protein levels were measured in Mock and p140Cap MDA-MB-231 cells, by quantitative RT-PCR or by ELISA on cell culture supernatants, respectively. Data are represented for n=3 experimental repeats, bar plot represented as mean ± SEM; two-tailed unpaired t test.

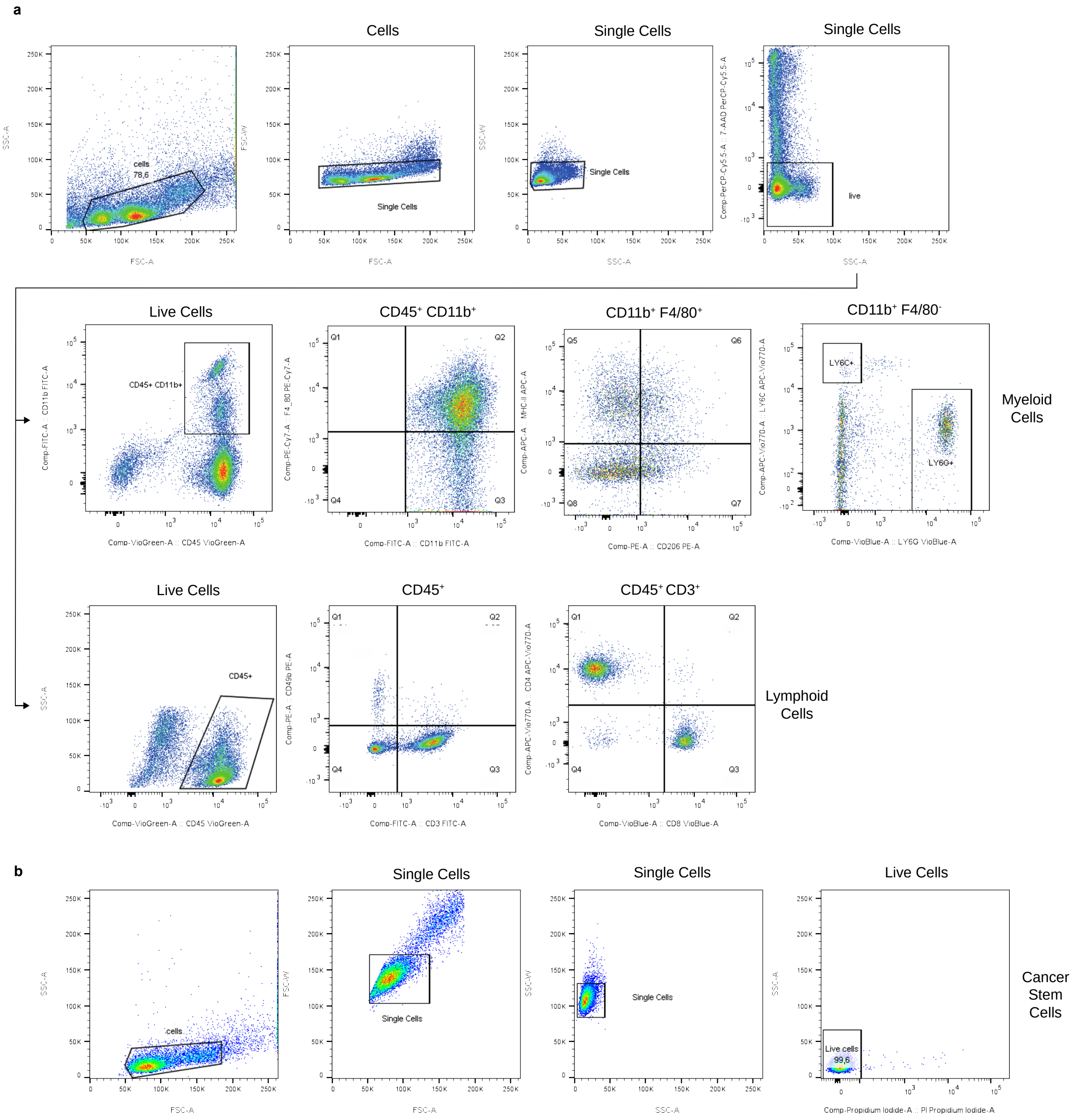

Supplementary Fig. 8

a Gating strategy for the tumor immune infiltrate in Figure 2 (panel **b-d**), Figure 4 (panel **a-b**), Figure 6 (panel **j**), Figure 9 (panel **f**)

b Gating strategy for the Cancer Stem Cell markers in Figure 5 (panel **d-e**).
